# A systematic review comparing the efficacy of 980 nm vs. 1470 nm wavelengths in laser hemorrhoidoplasty

**DOI:** 10.1007/s00384-024-04690-z

**Published:** 2024-07-24

**Authors:** Zhicheng Li, Jiong Wu, Nana Kwame Domme Brown, Philemon Kwame Kumassah, Kwabena Agbedinu, Peter C. Ambe

**Affiliations:** 1 Department of Coloproctology, Yueyang Hospital of Integrated Traditional Chinese and Western Medicine, Shanghai University of Traditional Chinese Medicine, Shanghai, 200437 China; 2 Department of Surgery, Korle Bu Teaching Hospital, Accra, Ghana; 3 Komfo Anokye Teaching Hospital, Kumasi, Ghana; 4 Department of Health, Chair of Surgery II, Witten/Herdecke University, Witten, Germany

**Keywords:** Laser hemorrhoidoplasty, LHP, Hemorrhoids, 1470-nm wavelength, 980-nm wavelength, Hemorrhoidal surgery

## Abstract

**Background:**

Laser Hemorrhoidoplasty (LHP) is a minimally invasive surgical option for the management of hemorrhoidal disease that has been increasingly adopted by surgeons over the last decade. Two wavelengths; 980 nm and 1470 nm have been employed in LHP. However, no data exist comparing the effects of these two wavelengths for this indication. This systematic review investigates both wavelengths for the management of hemorrhoids via the LHP procedure.

**Methods:**

This systematic analysis and meta-analysis was performed following the PICOS and PRISMA guidelines. A systematic research of MEDLINE, Scopus, Clinicaltrials.gov, Embase, Cochrane Central Register of Controlled Trials, CENTRAL and Google Scholar databases from inception until March 2024 was performed.

**Results:**

Overall, 19 studies including seven randomized control trials (RCT) and 12 non-randomized control trials with a total of 2492 patients were included in this systematic review and meta-analysis. The duration of LHP with both wavelengths was significantly shorter compared to open hemorrhoidectomy, postoperative pain and the rate of postoperative complications were significantly lower following LHP. There was no statistically significant difference in the rate of recurrence between LHP with the 980-nm wavelength and open hemorrhoidectomy. However, LHP with 1470-nm wavelength resulted in significantly higher recurrence rate compared to hemorrhoidectomy.

**Conclusion:**

Although no direct studies have compared the two wavelengths used in LHP, the outcomes of LHP seem to be independent of the wavelength used. Both wavelengths, when correctly used provide similar results, which are mostly better compared to open hemorrhoidectomy in terms of postoperative complications and postoperative pain, but not in terms of recurrence, where at least for the 1470-nm wavelength, LHP seems to show a higher recurrence rate when compared to open hemorrhoidectomy. Although a direct comparison of both wavelengths was not possible, technical issues regarding number of shots and energy per pile represent relevant parameters for recurrence after LHP.

## Introduction

Laser Hemorrhoidoplasty (LHP) describes a minimally invasive procedure for the management of symptomatic hemorrhoids using laser energy [1–4]. In the standard LHP, the laser energy is first applied just above the dentate line to coagulate the feeding vessels and thereafter into the engorged hemorrhoidal tissue causing its shrinkage and secondary adhesion onto the bowel wall [2, 5–7]. Because of its many advantages (e.g. small wounds at the puncture sites, low pain levels, outpatient procedure, early return to work, no continence disturbance), LHP has rapidly been adopted as a minimally invasive and tissue preserving technique in the management of hemorrhoidal disease [8–12]. While the results following LHP have been encouraging so far, there are still a lot of unanswered questions. Technically, two different wavelengths; 980 nm and 1470 nm have been employed by different users to perform LHP [13, 14]. The amount of energy applied to the hemorrhoidal tissue is directly proportional to both the wavelength of the laser used and the duration of application. This heterogeneity can also be easily identified amongst different users with regard to the amount of energy per pile and the number of piles treated [15]. Since both the efficacy and safety of laser-based surgery depends, at least partly, on the amount of energy, energy-correlated outcomes for laser surgery are a reasonable research topic. To the best of our knowledge, a direct comparison of the two wavelengths used in LHP has not been performed so far. Looking at the direct relationship between energy and outcome, studying this possible association may provide more insight to guide clinical practice. The aim of this study, therefore, was to investigate the two available laser wavelengths for LHP regarding postoperative outcomes.

## Methods

This study was conducted in strict accordance with the Cochrane Handbook of Systematic Reviews and Meta-analysis ver. 6.2 and reported according to the PRISMA (Preferred Reporting Items for Systematic Reviews and Meta-Analyses) statement guideline. A systematic literature review was conducted for articles published in Web of Science, Embase, Cochrane Library, PubMed. Articles were identified using search terms ‘hemorrhoids’ (Mesh term), ‘Laser’, ‘Open’, ‘Milligan-Morgan’, ‘Conventional’, ‘Excisional’, ‘Hemorrhoidectomy’ (Mesh term), and ‘Clinical Trial’ (Mesh term), and ‘1470 nm’, ‘980 nm’. This systematic review was registered with PROSPERO.

### Inclusion and exclusion criteria

We conducted a systematic review and meta-analysis of clinical trials and observational studies. Since no studies directly compared both wavelengths, the outcomes of each wavelength were compared with open hemorrhoidectomy. Thus, the meta-analysis was done on studies comparing LHP 1470 nm and 980 nm) against open surgery.

The inclusion criteria using the PICOS statement are as follows:

- P—patients undergoing management for hemorrhoidal disease.
- I—a group of patients who received LHP using either 980 nm or a1470 nm wavelength.
- C: a group of patients who underwent conventional hemorrhoidectomy.
- O: report at least one of the following results: operative time, complications, recurrence rate, postoperative day 1 pain using the visual analog pain (VAS) scale; (5)
- S: randomized or non-randomized clinical trials, observational studies (cohort or case–control) and case series; (6) full-text report (including preprint).

The exclusion criteria were as follows: (1) articles reported in languages other than English; (2) studies on stapler fixation; (3) no control studies; (4) unpublished studies or abstracts.

## Study selection and data extraction

**Fig. 1:**
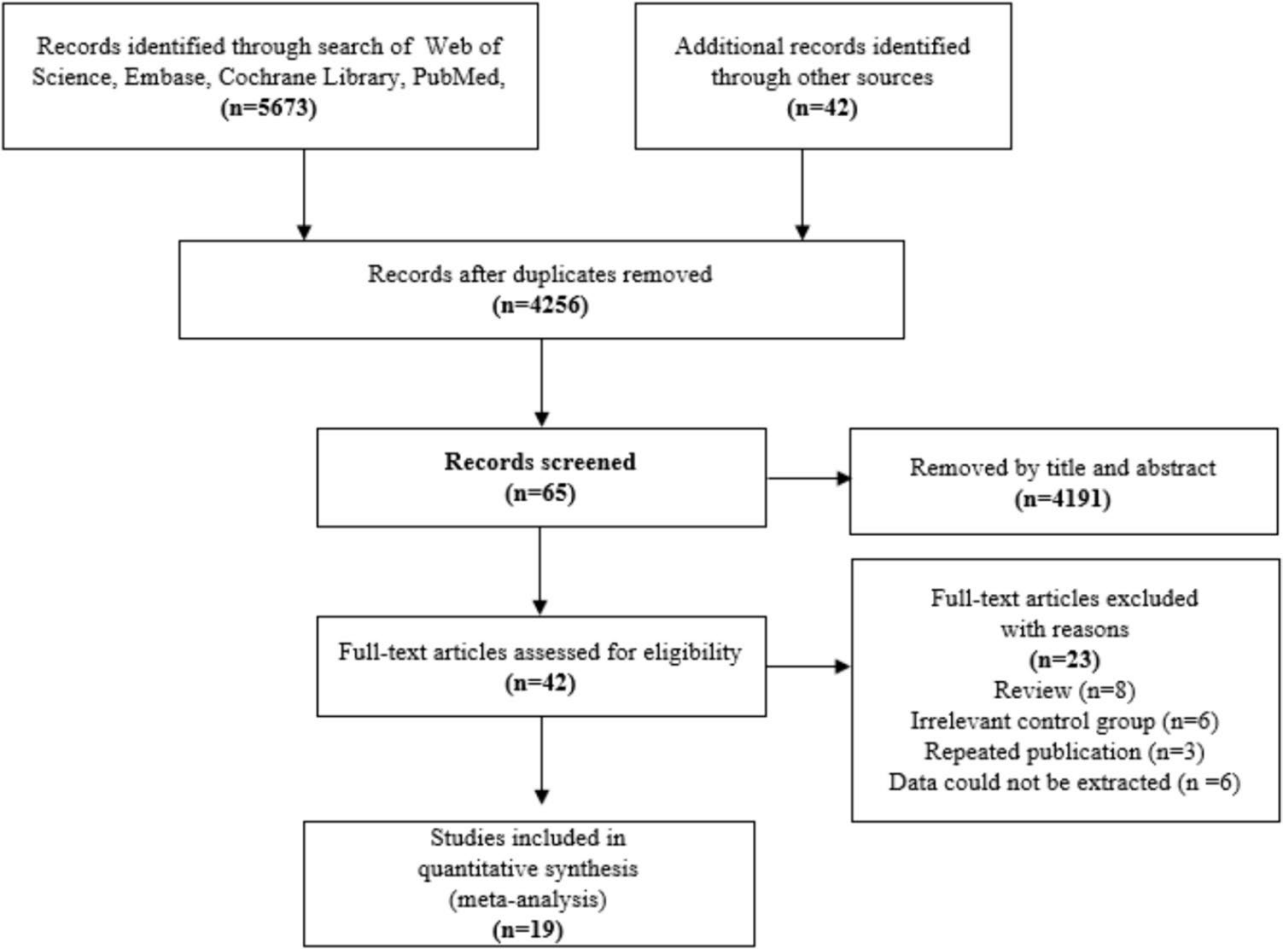
PRISMA flow chart for the selection of studies included in the study

Two independent investigators (ZL and JW) screened included articles according to the PRISMA guidelines for systematic reviews. Since no studies directly compared both wavelengths, the outcomes of each wavelength were compared with open hemorrhoidectomy. The initial step was to screen the title and abstract by two investigators to determine which articles met the criteria. Additionally, the references of eligible studies were hand-searched for more potential articles. The repeated articles were deleted. Finally, the full text is selected independently by two investigators, and the inconsistencies were resolved through discussion with PCA (Fig. 1).

### Statistical analysis

The RevMan 5.3 software provided by Cochrane Collaboration was used for data analysis. For dichotomous data, the risk ratio (RR) of 95% confidence interval (CI) was calculated, while for continuous data, average difference (MD) and standard average difference (95%CI) were calculated. Chi-square test and *I*^2^ statistics were used to evaluate the heterogeneity to determine whether the results of different studies were uniform.

## Results

Overall, 19 studies with 2492 patients were included in this article. The basic characteristics of these studies are revealed in Table 1.

**Table 1:** Characteristics of study participants from included studies

Author year	Period	Country	Pts(M/F) E C	Laser type	Main outcomes
Alsisy, 2019 [16]	April 2016 to September 2017	Egypt	18/12 15/15	980 nm	A B
Yahya, 2022 [17]	June 2020 to March 2021	Egypt	11/4 10/5	980 nm	A C
Mahmood, 2019 [18]	October 2014 to February 2018	Iraq	350/150 368/132	980 nm	D
Mert, 2022 [19]	December 2021 to May 2022	Turkey	9/11 11/39	980 nm	B D
Abdulkarim, 2020 [20]	2015 to 2018	Nairobi	11/10 9/6	980 nm	A B D
Nazari, 2015 [21]	January 2010 and 2011	Iran	19/10 12/18	980 nm	A C
Naderan, 2016 [13]	2011 and 2012	Iran	13/17 11/19	980 nm	A D
Mahmood, 2023 [22]	May 2020 and November 2021	Iraq	24/16 22/18	980 nm	B
Hassan, 2021 [23]	November 2019 and November 2020	Egypt	12/8 11/9	1470 nm	A B D
Gambardella, 2023 [24]	January 2018 to December 2019	Italy	81 93	1470 nm	A C
Maloku, 2014 [25]	January 2012 to June 2014	Kosova	11/9 12/8	980 nm	A
Maloku, 2019 [26]	June 2014 to May 2015	Montenegro	57/43 64/36	980 nm	A C
Cemil, 2024 [27]	September 2021 and October 2022	Turkey	38/11 22/9	1470 nm	A C
Durgun, 2023 [28]	January 2022 and June 2023	Turkey	17/31 22/30	1470 nm	A C D
Eskandaros, 2019 [29]	April 2017 till October 2019	Egypt	27/13 29/11	1470 nm	A B C D
Khan, 2021 [30]	January 2019 to June 2020	India	32/18 29/21	1470 nm	A B C
Tümer, 2023 [31]	April 2021 to April 2022	Turkey	47/38 72/46	1470 nm	A B
Verma, 2023 [32]	January 2020 to June 2021	India	24/6 17/13	1470 nm	A B
Poskus, 2020 [33]	April 2016 to April 2017	Lithuania	27/13 21/19	1470 nm	D

*A* Operative time, *B* Complications, *C* The VAS on postoperative day 1, *D* Recurrence rate

### Assessment of risk of bias

**Fig. 2:**
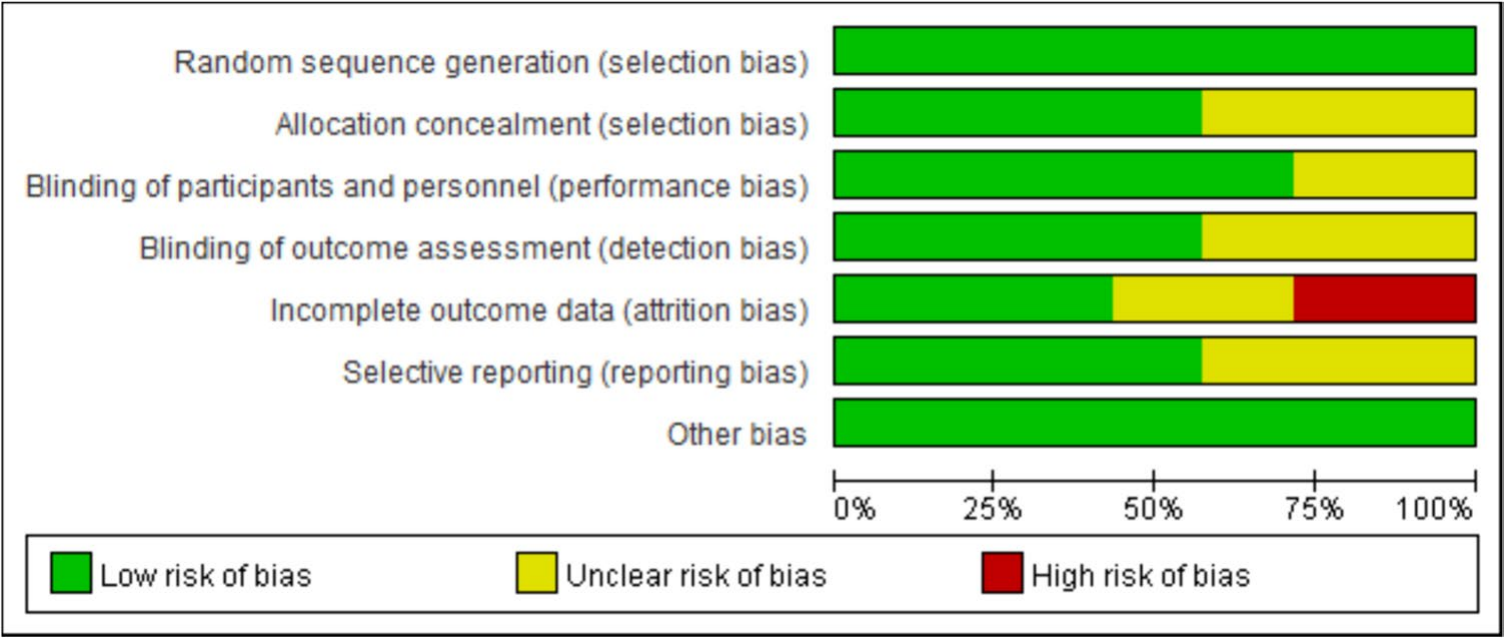
Assessment of risk of bias of RCTS

The quality of 7 RCTs was assessed by the Cochrane Handbook for Systematic Reviews and the 12 non-RCTs’ quality was assessed by the Newcastle–Ottawa Scale. The details and results are shown in Fig. 2 and Table 2.

**Table 2:** Assessment of risk of bias of non-RCTS

First author, year	Study design	Selection	Comparability	Outcome	Total score	Result
Alsisy, 2019	Cohort	***	**	***	8	Good
Yahya, 2022	Cohort	***	**	***	8	Good
Mahmood, 2019	Cohort	***	**	***	8	Good
Abdulkarim, 2020	Cohort	***	**	**	7	Good
Hassan, 2021	Cohort	***	**	***	8	Good
Gambardella, 2023	Cohort	***	**	***	8	Good
Maloku, 2014	Cohort	***	**	**	7	Good
Maloku, 2019	Cohort	***	**	**	7	Good
Durgun, 2023	Cohort	***	**	**	7	Good
Khan, 2021	Cohort	***	**	***	8	Good
Tümer, 2023	Cohort	***	**	***	8	Good
Verma, 2023	Cohort	***	**	***	8	Good

#### Operative time

**Fig. 3:**
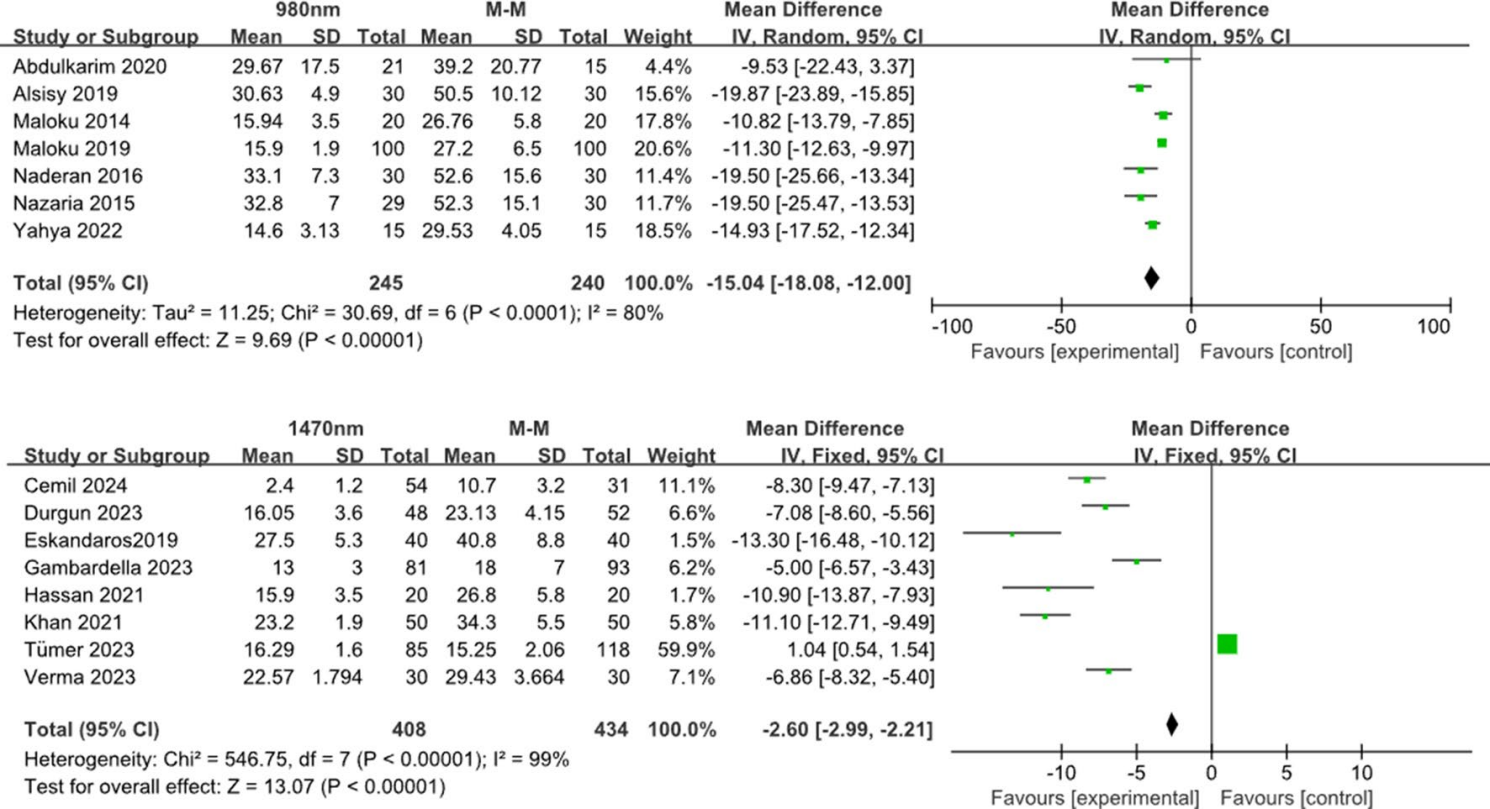
Forest plots for the length of surgery. Pooled analysis indicated LHP to be significantly faster compared to hemorrhoidectomy, irrespective of the wavelength used (**a** 980 nm, **b** 1470 nm)

The 980-nm group had a significantly shorter operative time (7 studies, *n* = 485) than the hemorrhoidectomy group (mean difference [MD], − 15.04; 95% CI, − 18.08 to − 12.00; *p* < 0.0001) (Fig. 3a, b).

The 1470-nm group also had a significantly shorter operative time (8 studies, *n* = 842) than the hemorrhoidectomy group (mean difference [MD], − 2.60; 95% CI, − 2.99 to − 2.21; *p* < 0.00001).

#### Complications

**Fig. 4:**
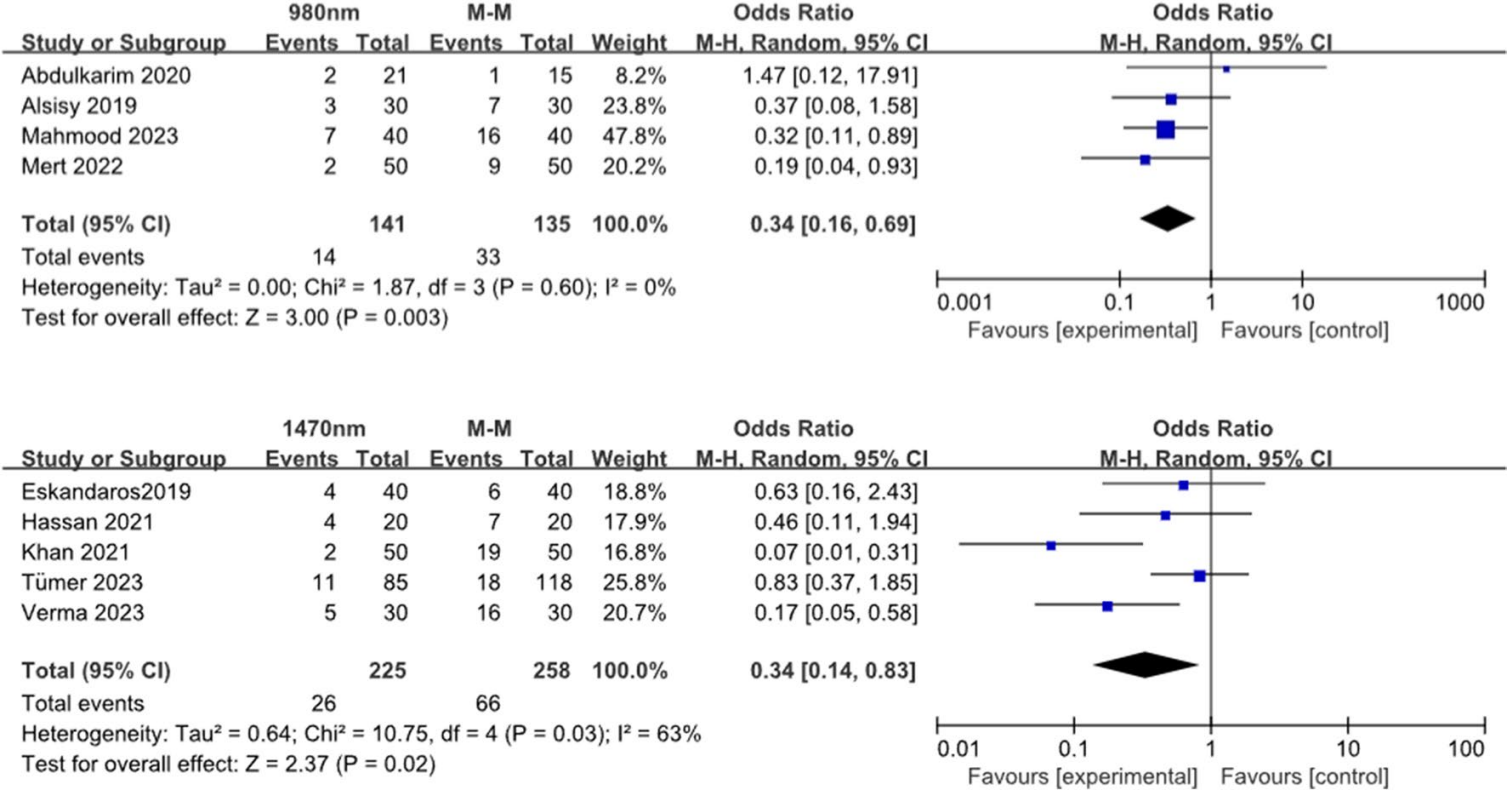
Forest plots for complication. The rate of postoperative complications (urinary retention, postoperative bleeding and acute postoperative thrombosis) was significantly lower in the LHP (irrespective of wavelength: **a** 980 nm, **b** 1470 nm) in comparison with open hemorrhoidectomy

There was significant difference in the rate of complications between the 980-nm group and the hemorrhoidectomy group of 4 studies (*n* = 276), with a OR of 0.34 (CI:0.16–0.69) *p* = 0.003 (Fig. 4a, b).

There was significant difference in the rate of complications between the 1470-nm group and the hemorrhoidectomy group from the pooled estimate of 5 studies (*n* = 483), with a OR of 0.34 (CI:0.14–0.83) *p* = 0.02.

#### The VAS on postoperative day 1

**Fig. 5:**
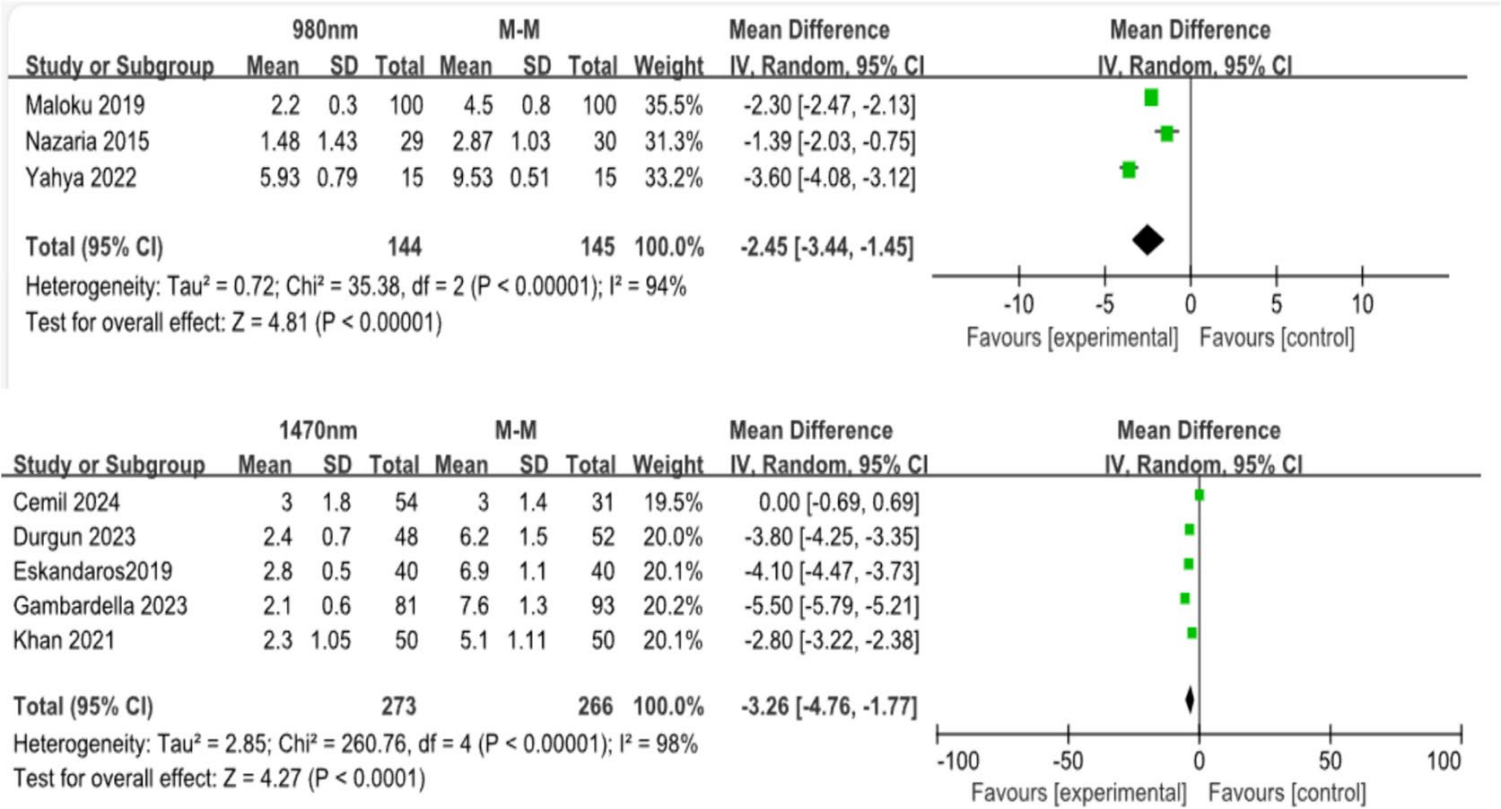
Forest plot for postoperative pain. Overall, postoperative pain was significantly lower following LHP (for both wavelengths **a** 980 nm, **b** 1470 nm) compared to hemorrhoidectomy

A total of three studies reported pain through the VAS on postoperative day 1 for the 980-nm wavelength with 289 patients. The LHP group had significantly reduced pain compared to the hemorrhoidectomy group with a mean difference of 2.45 (CI: 1.54–3.44, *p* < 0.0001) (Fig. 5a, b).

A total of five studies using 1470-nm wavelength reported pain through the VAS on postoperative day 1 with 539 patients. The LHP group had significantly reduced pain compared to the hemorrhoidectomy group with a mean difference of 3.26 (CI: 1.77–4.76, *p* < 0.0001).

#### Recurrence rate

**Fig. 6:**
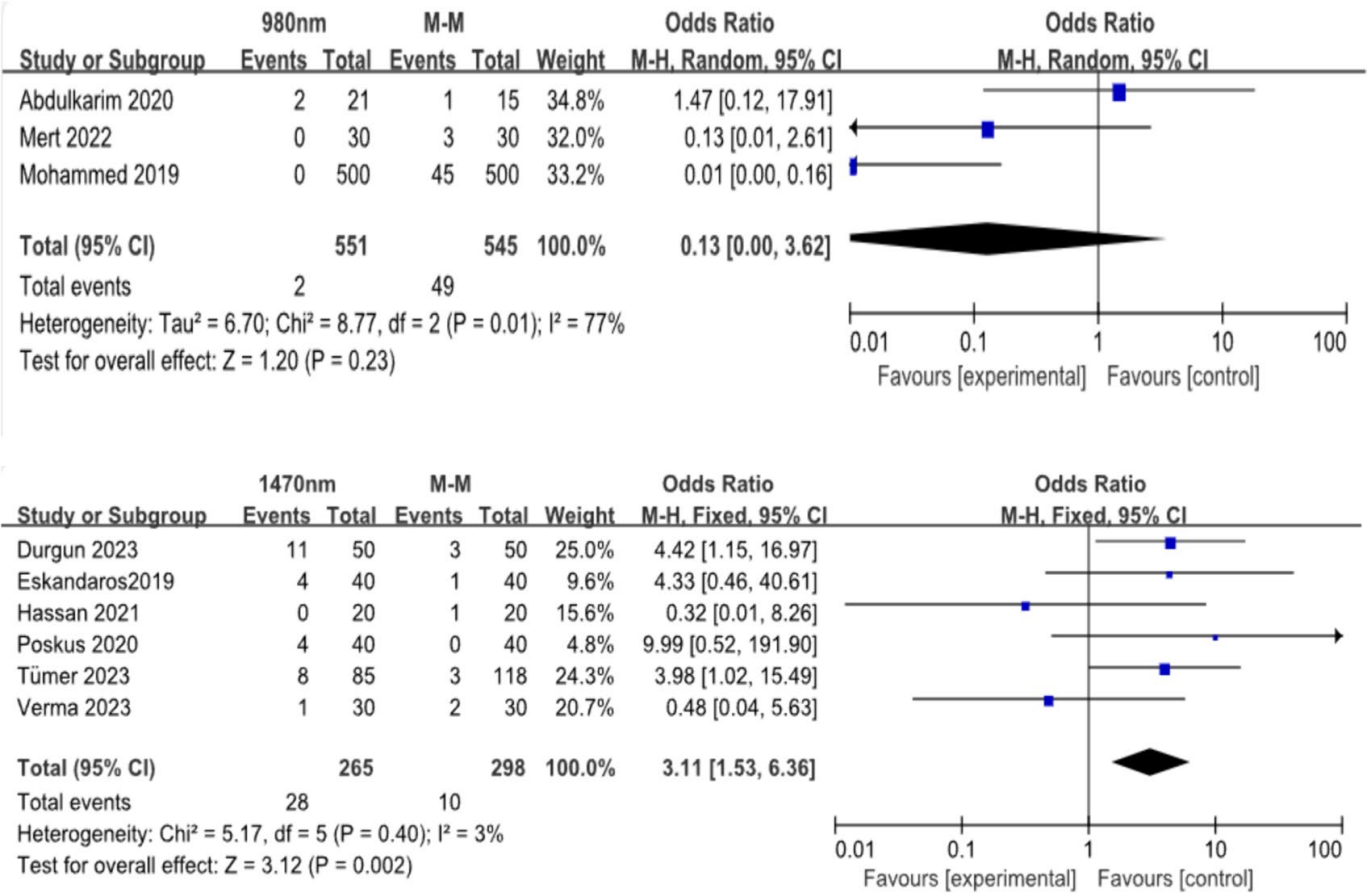
**a **and** b** rates of recurrence

Three studies with 1096 patients reported recurrence rates in the 980-nm group. There was no significant differences in the recurrence rates (OR:0.13; 95% CI, 0.00–3.62; *p* = 0.23) (Fig. 6a, b).

Six studies including 563 patients reported recurrence rates in the 1470-nm group. There was significant difference in the recurrence rates (OR:3.11; 95% CI, 1.53–6.36; *p* = 0.002). The recurrence rate of the open hemorrhoidectomy group was lower than that of the 1470-nm group.

As the follow-up time is not mentioned in some articles in the 980-nm laser group, and less data are included, further clinical study is needed.

## Discussion

The LHP procedure has been increasingly adopted over the last decade for the management of symptomatic hemorrhoids. Traditionally, two different wavelengths: 980 nm and 1470 nm have been used for this indication. However, no comparison has been made between both wavelengths to the best of our knowledge. Since the results of LHP is based on the direct effect of the laser energy applied to the piles, the outcomes of LHP therefore should partly depend on the wavelength used for treatment. Although this systematic review remains inconclusive due to the lack of studies investigating both wavelengths, pooled results confirm the efficacy and safety of both wavelength in performing LHP compared to open hemorrhoidectomy.

Alsisy et al. used the 980-nm wavelength to deliver the energy with a power of 15 W for 1.6 s (24 Joules) per shot in a study from Egypt [13]. In a study comparing hemorrhoidectomy with LHP Maloku et al. from Kosova used the 980-nm wavelength to deliver 13 W for 1.2 s per shot (18 J) [25]. In another study from Baghdad, Mahmood et al. used the 980-nm wavelength beginning with 8 W (which was increased as needed) for 3 s (minimum energy per shot 24 J) to perform LHP [22]. Despite the differences in energy employed in the above studies, the results of LHP in all three studies were similar.

The 1470-nm wavelength has been increasingly used more recently. As with the 980-nm wavelength, different amounts of energy have been used by different authors. Camil et al. for example used 8 W for 3 s per shot (24 J) [27]. While this setting has been used by many authors [24, 26], Khan et al. reported using 8.5 W without stating the duration of application to reach 150–350 J per segment [30]. Equally, little differences have been seen amongst these studies despite the differences in the amount of energy applied.

The minimally invasive nature of LHP is largely based on the means of access to the hemorrhoidal tissue by introducing the laser probe via a 2-mm puncture at level of the anal verge. LHP as a minimally invasive procedure has been shown to be a fast procedure compared to hemorrhoidectomy. This trend could be confirmed for 980-nm wavelength and for 1470-nm wavelength [34, 35]. Similarly, postoperative pain and overall complication rates were similar for both wavelengths in the LHP group, but significantly lower compared to the group managed with hemorrhoidectomy. Similar trends have been reported in the systematic analyses by Lakmal et al. [36] for the 980-nm wavelength as well as by Lie et al. and Tan et al. [34, 37].

An interesting finding from this systematic review was a difference in the rates of recurrence following LHP. While no statistically significant difference was observed between LHP using 980-nm wavelength and hemorrhoidectomy, the recurrence rate was significantly higher following LHP with 1470 nm compared to open hemorrhoidectomy. This finding, however, must be discussed regarding the surgical technique for LHP used in the included studies. In the study by Durgun and Yigit for example, the LHP procedure consisted of just five shot of 24 J per pile [28]. Haluk Tümer and Mevlut Agca also reported using 5–6 shots of 6–6.4 W [31]. The energy used in the LHP group by Eskandaros et al. was not clearly reported [29]. The same argument holds for the publication by Ahmed Hassan and Gamal Shemy [23] who stated 8 Watts for 7 s without reporting the number of shots. Finally, Verma et al. used 8 W for 1.6 s (12.8 J) [32]. Despite giving 8 shots per pile, the cumulative energy of 100.8 J was way below the expected energy needed to achieve a good LHP result. Looking at these technical variations, the piles in the LHP group in these studies were not adequately treated. The techniques used in these five studies about the number of shots and the amount of laser energy used are contrary to what is recommended in the standard LHP technique, i.e. 6–8 shots to manage the pile, resulting in 210–350 J.

A major limitation of this manuscript is the lack of studies with direct comparison of both wavelengths for LHP. Therefore, a direct analysis of pooled data to study the effect of the chosen wavelength was not possible. Also, there is a wide heterogeneity amongst users with regard to the technique of performing LHP that renders comparison of outcomes difficult [38]. Only studies in English language were included in this systematic review, thus there is a possibility that potentially relevant studies in languages other than English that may have affected the overall effects seen in this study were not included. More so, it is also possible that our search strategy may have missed some relevant studies.

Despites these limitations, our study clearly identifies a relevant gap in current literature about LHP and laser surgery in proctology in general. This gap may be of clinical relevance since the safety and efficacy of laser-based interventions in proctology are energy dependent and the amount of energy is obviously related to the chosen wavelength. Our study therefore may be instrumental in defining future research in this domain.

## Conclusion

Although no direct studies have compared the two wavelengths used in LHP, the outcomes of LHP seem to be independent of the wavelength used. Both wavelengths, when correctly used provide similar results, which are mostly better compared to open hemorrhoidectomy in terms of postoperative complications and postoperative pain, but not in terms of recurrence, where at least for the 1470-nm wavelength, LHP seems to show a higher recurrence rate when compared to open hemorrhoidectomy. Although a direct comparison of both wavelengths was not possible, technical issues regarding number of shots and energy per pile represent relevant parameters for recurrence after LHP.

This systematic review shows the need to standardize the LHP technique in order to achieve more homogenous results and better define the real role of LHP in the treatment of hemorrhoidal disease.

## Data Availability

All data used for this study have been included in the manuscript.
